# Periplasmic Binding Proteins in Thermophiles: Characterization and Potential Application of an Arginine-Binding Protein from *Thermotoga maritima*: A Brief Thermo-Story

**DOI:** 10.3390/life3010149

**Published:** 2013-02-05

**Authors:** Alessio Ausili, Maria Staiano, Jonathan Dattelbaum, Antonio Varriale, Alessandro Capo, Sabato D'Auria

**Affiliations:** 1 Laboratory for Molecular Sensing, Institute of Protein Biochemistry, CNR, Via Pietro Castellino, 111, Napoli, 80131, Italy; E-Mails: a.ausili@ibp.cnr.it (A.A.); m.staiano@ibp.cnr.it (M.S.); a.varriale@ibp.cnr.it (A.V.); a.capo@ibp.cnr.it (A.C.); 2 Department of Chemistry, University of Richmond, Richmond, VA 23173, USA; E-Mail: jdattelb@richmond.edu

**Keywords:** ABC transporters, periplasmic binding proteins, arginine, structural stability, unfolding

## Abstract

Arginine-binding protein from the extremophile *Thermotoga maritima* is a 27.7 kDa protein possessing the typical two-domain structure of the periplasmic binding proteins family. The protein is characterized by a very high specificity and affinity to bind to arginine, also at high temperatures. Due to its features, this protein could be taken into account as a potential candidate for the design of a biosensor for arginine. It is important to investigate the stability of proteins when they are used for biotechnological applications. In this article, we review the structural and functional features of an arginine-binding protein from the extremophile *Thermotoga maritima* with a particular eye on its potential biotechnological applications.

## 1. Introduction

### 1.1. ABC Transporter System and Periplasmic Binding Proteins

ATP-binding cassette (ABC) transporters are a ubiquitous distributed transmembrane protein superfamily involved in the active translocation through the membrane of a big variety of substrates, including ions, sugars, vitamins, drugs, metabolic products, lipids, amino acids and polypeptides. ABC transporters are involved in a wide number of cellular processes and they utilize the energy obtained by the ATP hydrolysis to carry out their functions, while their dysfunction underlies a number of genetic diseases [1,2,3]. ABC proteins are formed by four domains that may be organized in several modes: two highly conserved cytoplasmic nucleotide-binding domains (NBDs) which bind and hydrolyze ATP driving the translocation of the bound ligand and two variable transmembrane domains (TMDs) consisting of α-helices that allow the translocation of solutes across the membranes and provide specificity [4,5]. ABC transporters can be divided into two main functional groups that differ significantly both in their functioning mechanism and in their structural organization, mainly of TMD region: importers that are involved in substrate up-taking and exporters that are responsible for the secretion of various molecules [6,7,8]. Importers, that are present only in prokaryotes, are often accompanied by additional high-affinity binding proteins that are localized in the periplasmic space of gram-negative bacteria. These proteins named periplasmic binding proteins (PBPs) interact and associate with extreme specificity and affinity with different substrates that are distributed to the appropriate ABC transporter anchored to the inner membrane. The mechanism of ligand uptake by the synergic action of importers and PBPs is well functionally characterized for the maltose uptake system of *E. coli* and the structure of the entire complex (MBP-MalFGK_2_) has also been described in atomic details [9]. PBPs can recruit a broad number of nutrients and they are also present in other bacteria without periplasm [10] and in archaea [11] constituting one of the largest and widely distributed protein family in bacteria and archaea. To this day, many structures of different wild-type PBPs have been solved at very high resolution, and in all cases, they show some similar peculiar characteristics. Typically, these binding proteins are monomers consisting of two well-defined globular domains joined by a hinge forming a groove between them that constitutes the ligand-binding site. This structure allows the proteins to assume two different conformations depending on the presence or the absence of the ligand (closed and open form respectively), indeed the two domains act like a clamp and move closer each other as a consequence of the ligand binding [12]. Despite of their different sizes that vary between 20 and 60 kDa and a relatively little sequence homology [13], all PBPs share not only the previously described three dimensional conformation but also a similar secondary structure. Each domain consists of a β-sheet core formed by five or six β-strands surrounded by helices and depending on number and order of the strands, two structural classes of PBP superfamily can be identified: the class I possess six β-strands in the order β_2_β_1_β_3_β_4_β_5_β_6_ and the two domains are linked by three peptide hinges, while the class II is formed by five strands that take the form of β_2_β_1_β_3_β_5_β_4_ with two connecting segments between the two domains [12,14,15]. Recently, a third class of PBPs has been identified which do not fall into any of the previously described structural groups and it is characterized by a single long helix hinge [16,17].

### 1.2. Arginine-Binding Protein from Thermotoga Maritima and Its Potential Applications

Arginine-binding protein (ArgBP) is a member of PBPs superfamily belonging to the class II and it is capable to bind to arginine with very high affinity [18] and a ligand-binding specificity only for arginine [19] or for arginine, ornithine and/or lysine [20,21]. Besides the extremely important role in regulation of intracellular arginine concentration, ArgBP as many other PBPs can be employed in a wide range of biotechnological applications [22], among them ArgBP could be used as sensitive element in biosensor systems for detection of arginine in urine, serum and blood. Indeed, high levels of arginine in bodily fluids is an important signal of enzyme arginase malfunctioning in urea cycle, a disorder that can lead to hyper-argininemia, an autosomal recessive disease caused by high concentration of arginine and ammonia in the blood with dramatic consequences to human health, especially in neonatal age [23,24]. Moreover, arginine is also the precursor for the synthesis of endothelium-derived nitric oxide by the enzyme nitric oxide synthase [25]. Nitric oxide is an important factor for the regulation of blood flow and pressure, therefore anomalous levels of arginine can be the cause of disorders related to nitric oxide synthesis that can lead to vascular diseases like atherosclerosis and hypercholesterolemia [26,27]. Hence, the possibility to get a real-time and continuous detection and monitoring of arginine levels could be fundamental for diagnosis and treatment of these disorders. A reagentless optical biosensor based on ArgBP fluorescence could be a cheap, easy and powerful tool with a broad range of applications. Indeed, several PBPs have already been studied and applied in fluorescence-based biosensors for the detection of other molecules such as glutamine and glucose. In these cases glutamine-binding protein and glucose/galactose-binding protein were proposed as biosensors for optical assay of glutamine and glucose, respectively, by exploiting the intrinsic characteristics of the wild-type proteins, or by introducing residues in specific locations in order to label the proteins with fluorescent dyes and/or to decrease the ligand affinity of the binding protein to physiologically relevant ranges [28,29,30]. In general, the peculiar characteristic of these binding proteins of assuming open or closed form depending on the ligand-binding with the consequent large conformational change may be exploited to monitor possible changes in fluorescence emission of the aromatic residues or the fluorescent probes expressly linked to the protein. For this reason and for the advantage of avoiding the secondary components production and the modification of the sensor itself, PBPs could be employed as good scaffolds in fluorescence biosensing applications. High affinity and specificity of binding to the ligands combined to high sensitivity of the fluorescent probe is a very important starting point for the development of optical biosensors. However, this is a necessary but not sufficient property for practical applications of the biosensors. High stability under a wide range of different environmental conditions is also required, and an effective partial solution for this problem is given by extremophiles. Indeed, the use of proteins from extremophilic organisms is often preferred to that of their mesophilic homologues, since this provides the essential robustness to the biosensor. Here, a thermostable arginine-binding protein from the extremophile *Thermotoga maritima* (TmArgBP) is described with a view to its potential application as a scaffold for the creation of a solid fluorescent biosensor. *T. maritima* is a hyper-thermophilic gram-negative eubacterium originally isolated from different geothermal heated marine sediments in Italy, Iceland and the Azores, and it can perfectly grow in a range of temperature from 55 up to 90 °C with an optimum of 80 °C [31]. Though a eubacterium, *T. maritima* shows a large number of archaeal gene homologues that reveals an occurred lateral gene transfer during the evolution, demonstrated by a high gene sequence similarity [32,33]. Some thermostable sugar-binding proteins from this microorganism have been already partially characterized and potentially they could also be employed in biosensing [34,35,36]. The genomic sequence of T. maritima contains several open reading frames (ORFs) annotated as putative periplasmic binding proteins [32], among which tm0593 encodes a protein with similar sequence to several polar amino acid-binding protein although from this homology it could not be possible to predict the specific cognate ligand for the protein. The protein was determined to be an arginine-binding protein with a very high affinity and specificity for arginine as cognate ligand [37]. Innovative experiments of nano-flow electrospray ionization mass spectrometry (nano-ESI-MS) showed that this PBP had a strong preferential selectivity for binding to arginine, this technique allowed to detect the formation of the protein-ligand complex in the presence of arginine as ligand, on the contrary the presence of other amino acids such as histidine or asparagine did not lead to the formation of any complex, in fact in these cases only the free protein was observed [37]. Hence, in order to measure the binding affinity to the arginine, surface plasmon resonance (SPR) experiments were performed and a dissociation constant (K_d_) of 20 μM was determined. This K_d_ value was also compared to that obtained using glutamine instead of arginine, that was 160 μM, since the high sequence homology between this arginine-binding protein and a glutamine-binding protein from *E. coli* [38], confirming that the protein had high affinity and specificity only for the amino acid arginine [37].

## 2. TmArgBP Structure

**Figure 1:**
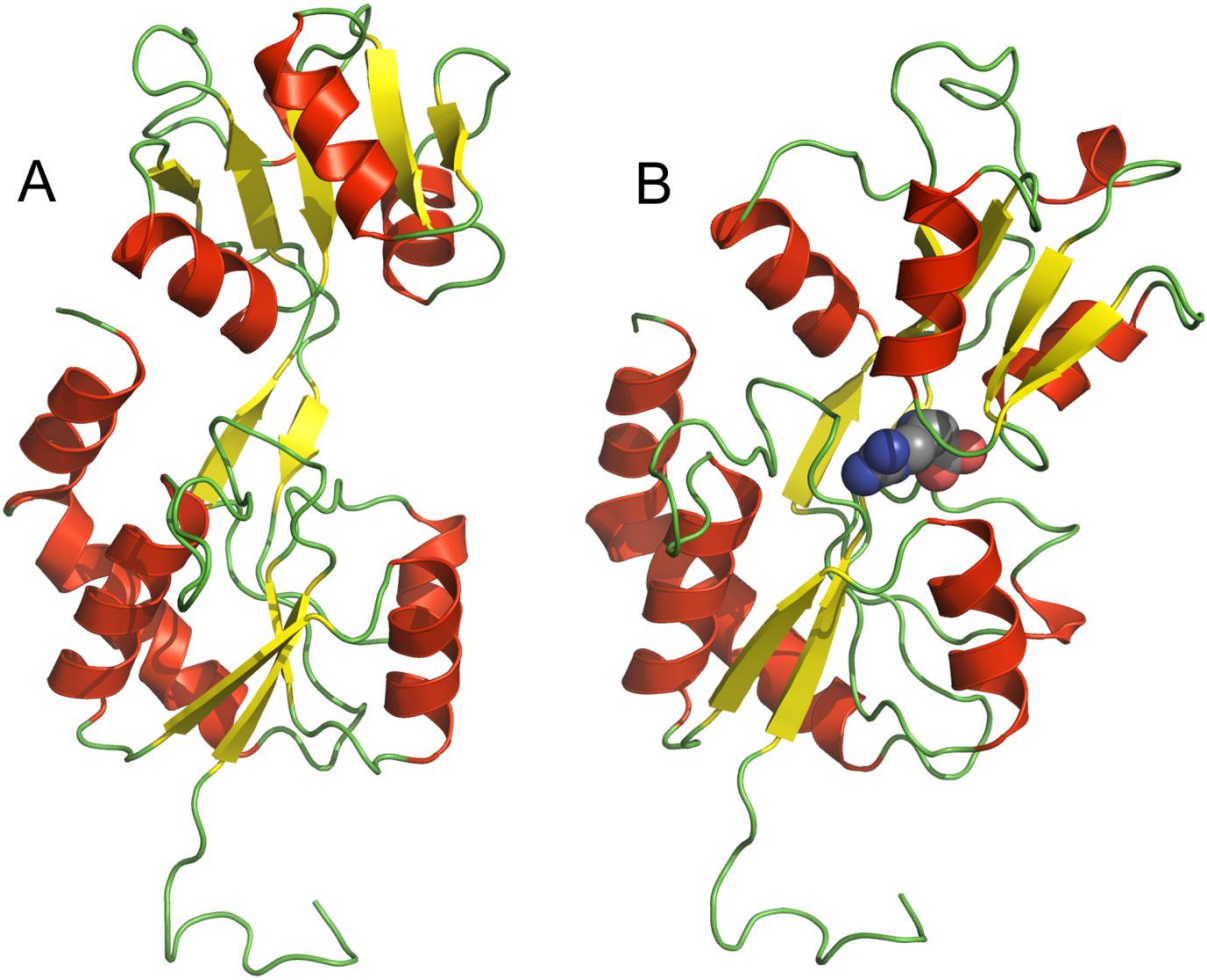
3D cartoon models of TmArgBP in the absence (**A**) and in the presence (**B**) of arginine (displayed in spheres). The ligand-binding induces the bending of the two domains that makes the protein assumes the closed form.

Having established that the protein encoded by *tm0593* was a specific arginine-binding protein (TmArgBP) with known dissociation constant, its molecular organization, conformational dynamics and structural stability were studied by means of different methods, including low-resolution biophysical techniques, preliminary X-ray crystallographic analysis, differential scanning calorimetry and molecular dynamics simulation. TmArgBP has a monomeric molecular weight of 27.7 kDa, unlike the most of PBPs, TmArgBP can exist as monomer, homodimer and homotrimer even under strongly denaturing condition [37]. The existence of oligomeric conformations was previously observed in other PBPs from *T. maritima* and also from other organisms and it seems to be related to the ligand transport and the protein membrane anchorage even if the precise specific function is still not well understood [39,40,41]. Since the three-dimensional structure of the TmArgBP monomer has not been already solved at high resolution, 3D protein models with and without the ligand were created by homology modeling with Modeller 9.5 (Figure 1 B and A, respectively) [42] using the structure of the ligand-bound Arg-, Lys-, His-binding protein from *G. stearothermophilus* (RMSD = 0.807 Å measured by InsightII – superimpose structure module) [43] and ligand-free Gln-binding protein from *E. coli* as templates (RMSD = 0.788 Å) [44] and as predictable, the TmArgBP models showed the characteristic PBP motif. In fact, TmArgBP monomer has the typical structure of PBPs family members, with a single polypeptide chain that folds into two domains connected by a hinge region. This conformation is strictly related to the ligand-binding function for all the PBPs, which is why even with a moderate level of sequence similarity they conserve this tertiary structure. In TmArgBP models, the single domains in open and closed forms were almost totally superimposable with a perfect coincidence of one of the two (the one containing both C- and N-terminal) with a RMSD of 0.66 Å, while the other domain showed a rotation of 50 °C around the hinge from the open to the closed conformation. While the binding to arginine induced the hinge-bending motion between the two domains, it had not any effect on the secondary structure. In both cases, the two domains conserved the typical β-sheet hydrophobic core surrounded by helices with no differences in secondary structures content, which was also confirmed by FTIR and CD experiments [42,45]. 

**Figure 2:**
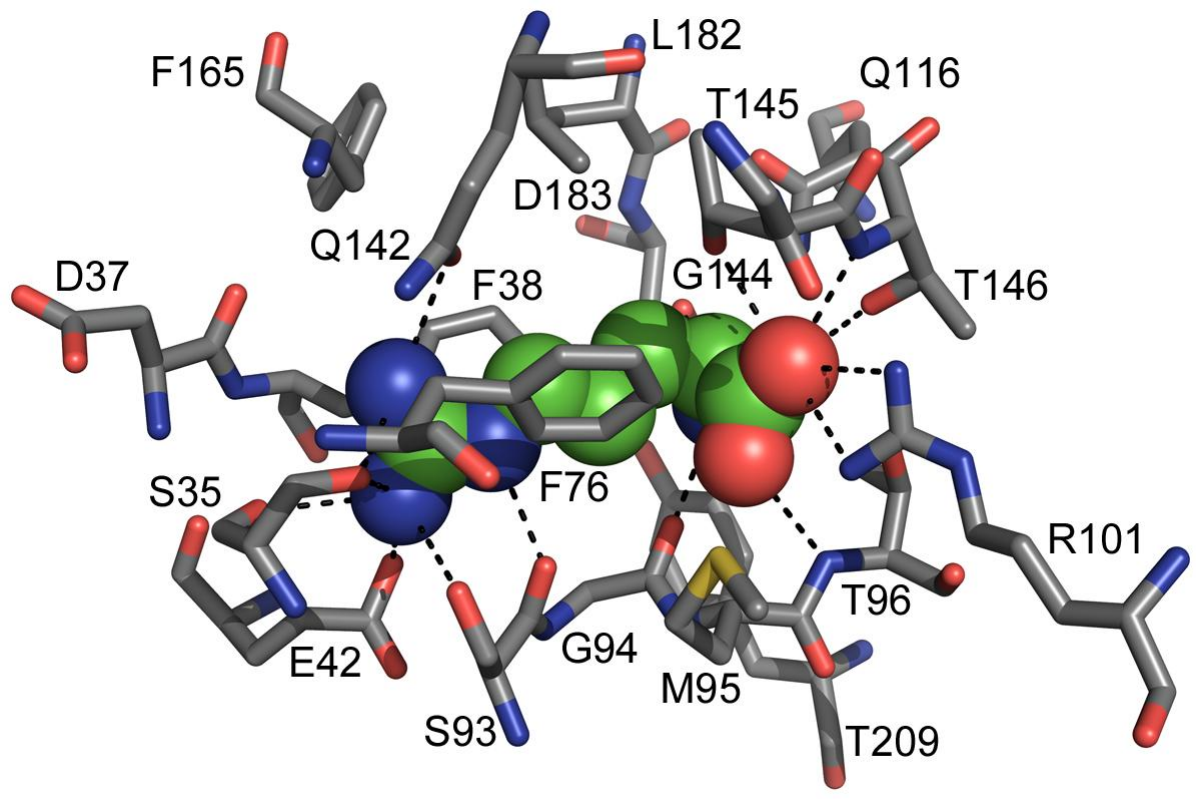
Close-up view of TmArgBP binding site with bound arginine. All the amino acidic residues lying within a distance of 5 Å from the ligand arginine are shown and represented in stick mode and labeled, while arginine is displayed in spheres. All the possible interactions that can be formed given charge, position and distance of each residue from the arginine are displayed in dashed lines.

A deeper molecular insight of the binding cleft of TmArgBP with bound arginine was provided by a visual analysis of the closed form model (Figure 2). The ligand-protein docking could involve several residues within 5 Å radius centered on the ligand arginine, but some of them appear to play a crucial role for the ligand docking to the binding site. In particular, E42, S35, S93 and Q142 are at a suitable distance to form H-bonds with the guanidine moiety of the ligand arginine. The residues D183 and G94 can interact with the amine group while T147, T146 and R101 form interactions with the carboxylic acid group. Finally, F38, F76 and T96 lie in a favorable position to create hydrophobic bonds with the arginine aliphatic straight chain.

Crystallization trials of both arginine-bound and arginine-free TmArgBP forms were also performed and some preliminary X-ray results were obtained [46]. In the presence of the ligand, ordered crystals of the protein were obtained in one day by using the detergent LDAO. The crystals presented a primitive hexagonal symmetry with an elongated unit-cell (a = 78.2, b = 78.2, c =434.7 Å) and the existence of three or four molecules per asymmetric unit. Otherwise, in the absence of arginine, ordered crystals suitable for X-ray diffraction experiments were obtained in one week by using PEG 3350 as precipitant. These crystals were orthorhombic with unit-cell parameters a = 51.8, b = 91.9, c = 117.9 Å and with the presence of two molecules per asymmetric unit. Arginine-bound and arginine-free TmArgBP crystals were diffracted to 2.7 and 2.25 Å resolution, respectively [46]. Trials to solve the structures of both protein forms at high resolution are in progress.

## 3. Stability of TmArgBP

As expected for proteins from extremophilic organisms, TmArgBP is endowed with an outstanding stability towards thermal and chemical denaturation. The complete protein thermal unfolding occurs around 120 °C while the melting temperature (T_m_) has been first estimated by Luchansky *et al.* [37] and then directly observed by Ausili *et al.* [45] at 115 °C.

The denaturation process is irreversible, one-step and cooperative, with a thermodynamic equilibrium model like:

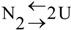

where N_2_ and 2U are the homo-dimeric native and the unfolded state, respectively. This model suggests that, despite TmArgBP exists as a dimer, and each monomer is formed by two well defined structural domains, only one energetic domain is present during the unfolding process [45]. Moreover, it was observed that the β-structure core is more stable than the more exposed α-helices and in particular, the three highly hydrophobic and completely buried β-strands of the central β-sheet are the most thermostable [42].

This strong protein stability under extremely high temperature conditions was also found in the presence of chemical denaturants such as SDS and guanidine hydrochloride (GdmCl) and at alkaline conditions. At 20 °C high concentrations of SDS (3.5% w/v) induce a partial loss of α-helices and β-sheet elements, however, the protein conserves a well-defined secondary structure that is progressively lost increasing the temperature up its complete unfolding at 95 °C [42]. A similar behavior was observed in the presence of GdmCl. The complete protein chemical denaturation can be achieved at 20 °C only in conditions of GdmCl saturation, while at 95 °C a concentration of at least 2.6 M GdmCl is required to fully denature TmArgBP [45]. At pH 10.5, the protein structure undergoes only little changes at 20 °C, in particular it is noted a partial lost of α-helix structures and a tertiary structure loosening. Moreover, at high pH values, the presence of two β-sheet populations located in the protein hydrophobic core is detectable during the denaturation process [42].

In any case, the cognate ligand binding exerts an important stabilizing effect on the protein structure. Indeed, the presence of arginine increases the T_m_ from 115 °C to 119 °C without affecting the overall unfolding mechanism, as well as it enhances its resistance to alkaline and chemical denaturant conditions [37,42,45].

## 4. Tryptophan Microenvironment

**Figure 3:**
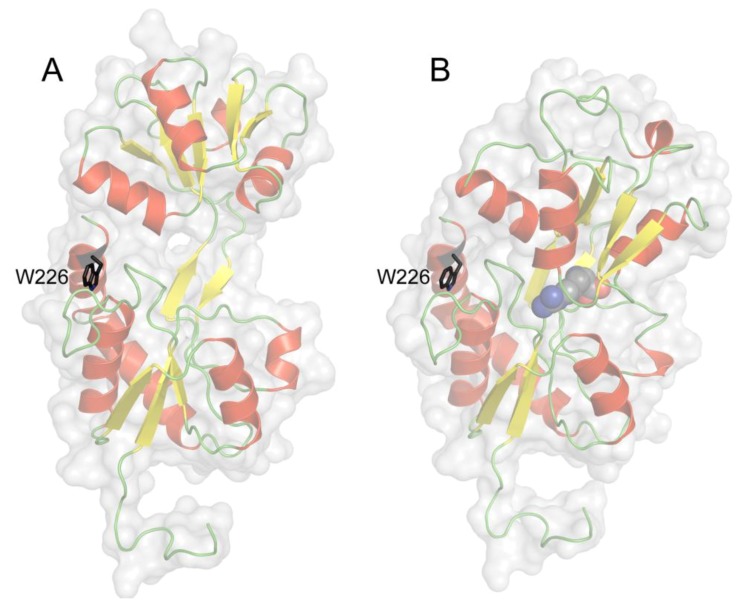
Position of Trp^226^ in TmArgBP. The indole side chain is in evidence, displayed in sticks and labeled. Panel A and B depict the open and closed form, respectively. The residue is far from the binding site and enough buried from the aqueous interface to avoid the direct exposition to the solvent. Notice that the presence of the ligand (displayed in spheres) does not affect the Trp^226^ position.

**Figure 4:**
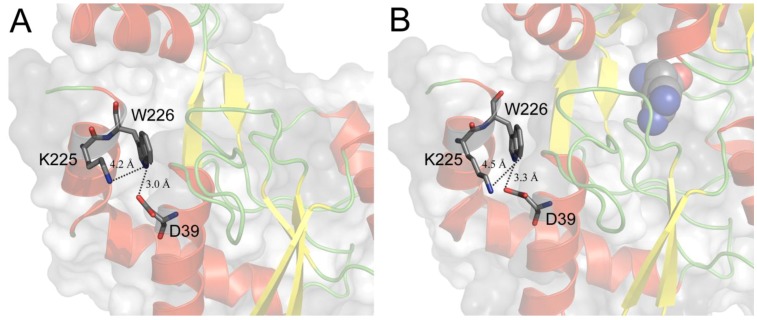
Close-up view of Trp^226^ microenvironment. Trp^226^ and the residues that possibly interact with it are in evidence, displayed in sticks and labeled. The black dashed lines represent the distances between the functional groups involved in the Trp^226^ stabilizing interactions. A and B show the tryptophan microenvironment for open and closed form, respectively. The interaction distances, labeled in the figure, remain roughly unvaried.

In TmArgBP only one tryptophan residue (Trp^226^) is present and it is localized in the α-helix at C-terminal of the protein at the opposite side of the binding site (Figure 3). Trp^226^ results to be stabilized by electrostatic interactions with near residues. In particular it is possible to notice the presence of stabilizing interactions between the carboxyl group of Asp^39^ facing the positive end of the Trp^226^ indole dipole (N1) (atomic distance = 3.0 Å in the open form) and between the positive charge of Lys^225^ facing the negative end of the Trp^226^ dipole (C4–C5) (atomic distance = 4.2 Å in the open form) (Figure 4A). Trp^226^ environment is highly homogeneous, polar and rather rigid, while the tryptophan indole ring is largely but superficially buried within the protein fold. In fact, the indole ring is not in direct contact with the aqueous phase, but it is very near to it (< 0.5 Å). Moreover, the region of Trp^226^ is not involved in the association of the subunits and also that monomer formation has no significant impact on the protein structure near the indolic residue [45]. Finally, Trp^226^ environment not only is far from the binding site but also its local structure/dynamics is totally disconnected from the binding site region. That was both observed from the analysis of Trp^226^ position and interactions involving the Trp^226^ microenvironment in the predicted structures illustrated in Figure 3, Figure 4, and inferred from the absolute absence of any change in fluorescence and phosphorescence tryptophan intrinsic emission when the protein assumes the closed form in the presence of the ligand [37,45]. This phenomenon is quite unusual for proteins, which undergo evident conformational changes like the PBPs and in our best knowledge this is the first protein that does not show changes in phosphorescence emission upon ligand binding.

## 5. Conclusions

TmArgBP could be employed in several biotechnological applications including optical biosensing for arginine detection based on fluorescence. Unfortunately, the possibility of using the intrinsic Trp^226^ emission for this purpose has to be discarded in TmArgBP, but its extremely advantageous characteristics of structural stability and selectivity/sensibility for arginine-binding could make this protein a potential candidate for the design of such a biosensor. Attempts to produce new mutant proteins aimed at providing to TmArgBP an optical sensitivity for conformational changes due to arginine binding are currently in progress with attractive outlooks. The idea is to create double mutants inserting reactive residues in order to attach fluorescent dyes in advantageous positions. The location of the mutations will allow the approach of the dyes when the protein assumes the closed form in the presence of arginine that will be detectable by means of RET or PET techniques. A biosensor based on this methodology would avoid many disadvantages of traditional arginine-detection systems such as high costs, response slowness and laborious sample preparations by qualified operators. For these reasons, we are confident that the design of a fluorescence-based biosensor would be an important advance in arginine detection.
